# Rising fluoroquinolone resistance in *Campylobacter* isolated from feedlot cattle in the United States

**DOI:** 10.1038/s41598-017-00584-z

**Published:** 2017-03-29

**Authors:** Yizhi Tang, Orhan Sahin, Nada Pavlovic, Jeff LeJeune, James Carlson, Zuowei Wu, Lei Dai, Qijing Zhang

**Affiliations:** 1 Department of Veterinary Microbiology and Preventive Medicine, Ames, IA USA; 2 Department of Veterinary Diagnostic and Production Animal Medicine, Ames, IA USA; 3 Food Animal Health Research Program, Ohio State University, Wooster, OH USA; 4 National Wildlife Research Center, USDA APHIS, Fort Collins, CO USA

## Abstract

Antibiotic resistance, particularly to fluoroquinolones and macrolides, in the major foodborne pathogen *Campylobacter* is considered a serious threat to public health. Although ruminant animals serve as a significant reservoir for *Campylobacter*, limited information is available on antibiotic-resistant *Campylobacter* of bovine origin. Here, we analyzed the antimicrobial susceptibilities of 320 *C. jejuni* and 115 *C. coli* isolates obtained from feedlot cattle farms in multiple states in the U.S. The results indicate that fluoroquinolone resistance reached to 35.4% in *C. jejuni* and 74.4% in *C. coli*, which are significantly higher than those previously reported in the U.S. While all fluoroquinolone resistant (FQ^R^) *C. coli* isolates examined in this study harbored the single Thr-86-Ile mutation in GyrA, FQ^R^ *C. jejuni* isolates had other mutations in GyrA in addition to the Thr-86-Ile change. Notably, most of the analyzed FQ^R^ *C. coli* isolates had similar PFGE (pulsed field gel electrophoresis) patterns and the same MLST (multilocus sequence typing) sequence type (ST-1068) regardless of their geographic sources and time of isolation, while the analyzed *C. jejuni* isolates were genetically diverse, suggesting that clonal expansion is involved in dissemination of FQ^R^ *C. coli* but not *C. jejuni*. These findings reveal the rising prevalence of FQ^R^ *Campylobacter* in the U.S. and provide novel information on the epidemiology of antibiotic-resistant *Campylobacter* in the ruminant reservoir.

## Introduction

*Campylobacter* is a leading cause of bacterial foodborne gastroenteritis worldwide and is a major public health problem^1, 2^. Although the majority of *Campylobacter* infections are self-limited and do not require antimicrobial treatment, antibiotics are indicated for severe and chronic conditions^3^. Clinical treatment of campylobacteriosis requires the use of fluoroquinolone (FQ) or macrolide antibiotics. However, antibiotic-resistant *Campylobacter* is becoming increasingly prevalent. Due to the rising resistance, especially to FQ, the Centers for Disease Control and Prevention (CDC) has recently identified drug-resistant *Campylobacter* as a serious antibiotic resistance threat in the United States^4^. The CDC reported that almost 25% of human *Campylobacter* isolates were resistant to ciprofloxacin in the USA^4^. Development and transmission of antibiotic resistant *Campylobacter* is complicated by the fact that *Campylobacter* is a zoonotic pathogen and is exposed to antibiotics used in both animal production and human medicine.

Contaminated poultry meat is frequently recognized as the major source for human infections^5^. However, ruminants also play a significant role in epidemiology of human *Campylobacter* infections and are increasingly reported as the implicated source^6–9^. Ruminant *Campylobacter* contributes to human disease via multiple transmission routes including direct contact (e.g. petting zoo and occupational exposure), consumption of unpasteurized milk (and associated dairy products), and environmental contamination (e.g., water and produce)^10–12^. Molecular typing methods, such as multilocus sequence typing (MLST) and pulsed field gel electrophoresis (PFGE), revealed that certain genotypes of *C. jejuni* from ruminants are indistinguishable from human isolates^12–14^, linking ruminant *Campylobacter* to human diseases. Raw milk is a well-recognized transmission route as a number of raw milk associated outbreaks of campylobacteriosis have been documented^15–18^. Ruminant *Campylobacter* may also contaminate water supplies via agricultural runoff. A waterborne outbreak associated with *Campylobacter* was reported to be the result of contamination of the town’s water supply with *Campylobacter* originating from a cattle farm in the vicinity^11^. Thus, control of *Campylobacter* in ruminants will have a direct impact on food safety and human health.

Despite the importance of ruminant *Campylobacter* in foodborne disease, few studies have been conducted to understand antibiotic-resistant *Campylobacter* from cattle. Earlier reports from the U.S. (including the Feedlot 1999 and Dairy 2002 NAHMS studies) and Canada indicated very low levels of FQ resistance (less than 5%) in *Campylobacter* isolates from cattle^19–22^. Bae *et al*.^23^ also reported a low level (ca. 5%) of resistance to ciprofloxacin in *C. jejuni* from different cattle production types in Western U.S., although *C. coli* isolates from the same study had much higher (ca. 45%) resistance rate to this drug during 2002–2003. Similarly, a study on *Campylobacter* from dairy cattle in the Midwest U.S. during mid-2000s indicated that less than 1% of isolates were resistant to ciprofloxacin^24^. However, a slaughterhouse survey^25^ conducted during late 2008 in the U.S. found that high percentage of both *C. jejuni* and *C. coli* (27.3% and 49.2%, respectively) from different types of cattle types (including both feedlot cattle and adult cows and bulls) were resistant to ciprofloxacin.

These observations point to a possible rising trend of FQ-resistance in the U.S. and highlight the need for conducting surveillance studies on a national scale to assess antibiotic resistance in ruminant *Campylobacter*. Although the National Antimicrobial Resistance Monitoring System (NARMS) operated by USDA monitors the occurrence of antimicrobial resistance in *Campylobacter* isolates from food animals at slaughter, the sampling and testing strategy does not include cattle and is limited to chicken carcass rinsates (http://www.ars.usda.gov/Main/docs.htm?docid=6750&page=2). To understand the ecology and facilitate control of antimicrobial resistant *Campylobacter* in the ruminant reservoir, we determined in this study the antimicrobial susceptibility of *Campylobacter* isolates derived from feedlot cattle operations in geographically diverse regions in the U.S.

## Results

### Prevalence of *Campylobacter* in feedlot cattle

The overall prevalence rate of *Campylobacter* in the feedlot cattle feces was 72.2% (2298/3184), and ranged between 69.2–78.2% among the different states from which the samples were derived. Of the *Campylobacter* isolates, 82.1% (1886/2298) were identified as *C. jejuni*, and 15.0% (344/2298) were determined to be *C. coli* by PCR (Fig. S1). The remaining 68 isolates (2.9%) were of different *Campylobacter* spp. than *C. jejuni* and *C. coli* and were not characterized further to species level (Table 1).

**Table 1:** *Campylobacter* species isolated from fecal samples of feedlot cattle from five states in the U.S.

State	Feedlot herds	Cattle samples	Positive cattle (%)	*C. jejuni* (%)	*C. coli* (%)
Iowa	8	800	554 (69.2)	487 (87.9)	56 (10.1)
Texas	8	576	414 (71.9)	367 (88.6)	35 (8.5)
Missouri	3	300	210 (70.0)	191 (91.0)	16 (7.6)
Colorado	8	758	593 (78.2)	438 (81.5)	124 (20.9)
Kansas	8	750	527 (70.3)	403 (76.5)	113 (21.4)
Total	35	3184	2298 (72.2)	1886 (82.1)	344 (15.0)

### Antimicrobial susceptibility of the *C. jejuni* and *C. coli* isolates from feedlot cattle

Of the 320 representative cattle *C. jejuni* isolates selected across the 35 feedlots tested in this study, 281 (88.1%) were found to be resistant to tetracycline, 114 (35.6%) were resistant to ciprofloxacin, and 110 (34.3%) were resistant to nalidixic acid. Resistance to azithromycin, clindamycin, erythromycin, florfenicol, gentamicin and telithromycin was low (one isolate for each) (Table 2). Among the 115 representative cattle *C. coli* isolates tested, 86 (74.8%) were found to be resistant to tetracycline, 89 (77.4%) were resistant to ciprofloxacin, 95 (82.6%) were resistant to nalidixic acid, and 5 (4.3%) were resistant to florfenicol and clindamycin. None of the *C. coli* isolates were resistant to azithromycin, erythromycin, gentamicin or telithromycin (Table 2). The ciprofloxacin resistance in *C. coli* (77.4%) was significantly (*P* < 0.05) higher than in *C. jejuni* (35.6%), as was the resistance rate for nalidixic acid (82.6% vs. 34.3%), whereas resistance to tetracycline was comparable (74.8% vs. 88.1%) between *C. coli* and *C. jejuni* (*P* > 0.05), respectively (Table 2). The resistance rates of either *C. jejuni* or *C. coli* isolates for tetracycline, ciprofloxacin and nalidixic acid did not vary substantially among different states (Data not shown). These results indicated an overall high rate of FQ resistance in feedlot cattle *Campylobacter* isolates, especially in *C. coli*.

**Table 2:** Prevalence of antimicrobial resistance in *Campylobacter jejuni* (n = 320) and *C. coli* (n = 115) from feedlot cattle.

Antibiotics	Range tested (μg/ml)	Resistance breakpoint (μg/ml)	No. (%) of resistance in cattle	
			*C. jejuni*	*C. coli*
Azithromycin	0.015–64	≥8	1 (0.3)	0
Ciprofloxacin	0.015–64	≥4	114 (35.6)	89 (77.4)*
Clindamycin	0.03–16	≥8	1 (0.3)	5 (4.3)
Erythromycin	0.03–64	≥32	1 (0.3)	0
Florfenicol	0.03–64	≥16	1 (0.3)	5 (4.3)
Gentamicin	0.12–32	≥8	1 (0.3)	0
Nalidixic acid	4.0–64	≥32	110 (34.3)	95 (82.6)*
Telithromycin	0.015–8	≥16	1 (0.3)	0
Tetracycline	0.06–64	≥16	281 (88.1)	86 (74.8)

*Significantly different resistance (*P* < 0.05) compared with *C. jejuni*.

Multiple drug resistance in *C. jejuni* and *C. coli* from cattle was observed frequently. Of the 320 *C. jejuni* isolates tested, 114 (35.6%) were resistant to two or more antimicrobial agents, 100 (31.2%) were resistant to three or more antibiotics, 3 were resistant to four or more agents, 2 were resistant to five or more drugs, and 1 was resistant to seven antibiotics including azithromycin, ciprofloxacin, erythromycin, gentamicin, tetracycline, nalidixic acid and telithromycin (Table 3). None of the *C. jejuni* isolates were resistant to all nine drugs tested. Of the 115 *C. coli* isolates, 89 (77.3%) were resistant to two or more antimicrobial agents, 63 (54.7%) were resistant to three or more drugs, and 5 (4.3%) were resistant to five antibiotics (Table 3). None of the *C. coli* isolates were resistant to six or more antimicrobial agents included in the MIC test. Only one *C. jejuni* isolate was co-resistant to both ciprofloxacin and erythromycin, while none of the *C. coli* isolates displayed co-resistance to these two antibiotics (Table 3). The most common multidrug resistance pattern was to ciprofloxacin, nalidixic acid, and tetracycline, which was observed in ~30% of *C. jejuni* and 50% of *C. coli* isolates, respectively (Table 3).

**Table 3:** Multidrug resistance patterns among *C. jejuni* and *C. coli* isolates from feedlot cattle.

Antimicrobial agent	*C. jejuni* (n = 320)	*C. coli* (n = 115)	Total (n = 435)
TN	2 (0.6%)	4 (3.5%)	6 (1.4%)
CNT	97 (30.3%)	58 (50.4%)	155 (35.6%)
CNTG	1 (0.3%)	0	1 (0.2%)
CNTFL	1 (0.3%)	5 (4.3%)	6 (1.4%)
CNTAEI	1 (0.3%)	0	1 (0.2%)

A, Azithromycin; C, Ciprofloxacin; E, Erythromycin; T, Tetracycline; F, Florfenicol; N, Nalidixic acid; I, Telithromycin; L, Clindamycin.

### Genetic diversity of FQ^R^*Campylobacter* from cattle

**Figure 1:**
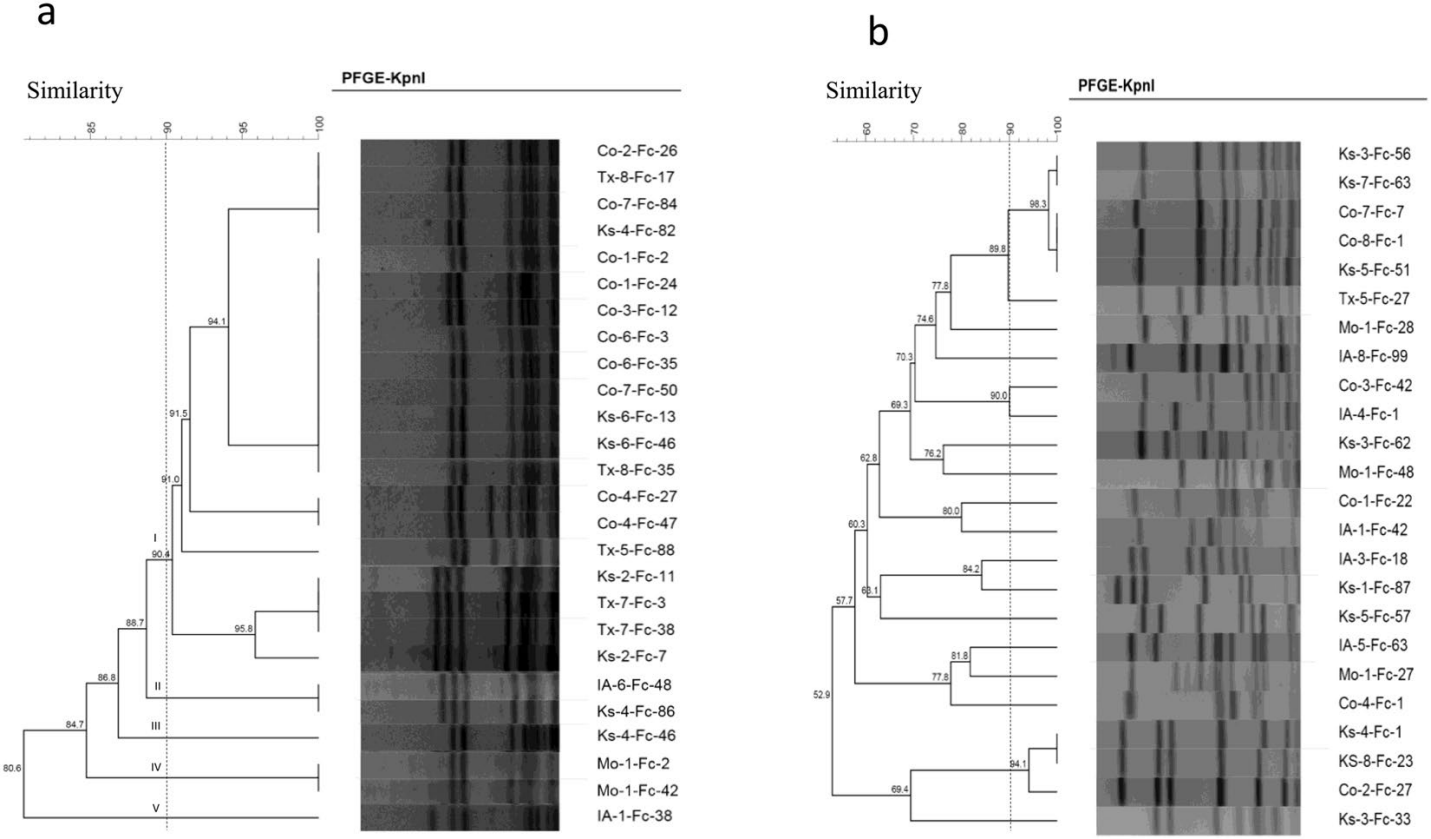
Dendrogram constructed using the PFGE patterns of *Kpn*I digested FQ^R^ *C. coli* (Fig. 1a) and *C. jejuni* (Fig. 1b) isolates. Numbers of bootstraps represent similarity. Clusters are determined using a cut off of 90% similarity. Isolate names are listed on the right side of each dendrogram. Tx: Texas; Co: Colorado; IA: Iowa; Ks: Kansas; Mo: Missouri; Fc: Feces.

To determine if FQ^R^ *Campylobacter* isolates are genetically related, we analyzed the genetic diversity of representative isolates using PFGE and MLST. A total of 26 FQ^R^ *C. coli* isolates were randomly selected for this purpose. Based on the 90% similarity level, the 26 isolates were grouped into five separate clusters, with the vast majority of isolates (76.9%, 20/26) grouped in cluster I, two isolates in cluster II and IV and one isolate in each of the remaining two clusters (Fig. 1a). The first four clusters had closely related PFGE profiles; the only noticeable difference among the patterns was the presence of extra one or two bands in some isolates (Fig. 1a). The 26 *C. coli* isolates tested came from 15 different feedlot cattle farms in five different states that were sampled at different times. The 20 strains that grouped together in cluster I included those from 11 different farms in three different states, 10 from Colorado, 6 from Kansas, 4 from Texas while those in Cluster II were from 2 different farm in two states, the two isolates in Cluster IV came from the same farm in Missouri. MLST showed that the isolates grouped in cluster I, II, III and IV had the same sequence type (ST-1068), while the isolate in cluster V was ST-5446.

Additionally, 24 FQ^R^ *C. jejuni* isolates were examined for genetic diversity via PFGE. In contrast to the situation with *C. coli*, no predominant genotypes were observed among the *C. jejuni* isolates. There were a total of 17 different PFGE profiles using 90% similarity as the cut off (Fig. 1b). MLST analysis of 7 *C. jejuni* isolates (Ks-3-Fc-56, Co-4-Fc-1, Co-1-Fc-22, Ks-4-Fc-1, IA-5-Fc-63, IA-3-Fc-18, Mo-1-Fc-27) representative of different PFGE pattern showed 6 different sequence types (ST982, ST3855, ST219, ST45, ST6751, ST459, ST3855 respectively). The MLST result confirmed the genetic diversity of FQ^R^ *C. jejuni* isolates. All together the PFGE and MLST findings suggest that clonal expansion is not involved in dissemination of FQ^R^ *C. jejuni* on the cattle farms.

### Antibiotic resistance mechanism of FQ^R^*Campylobacter*

In *Campylobacter*, FQ resistance is conferred by point mutations in the *gyrA* gene in conjunction with the function of the CmeABC efflux pump^26^. To examine the mechanisms of FQ resistance, the quinolone resistance determining region (QRDR) in *gyrA* of 27 FQ^R^ *C. coli* and 27 FQ^R^ *C. jejuni* isolates were sequenced to determine the mutations associated with FQ resistance. These isolates were selected to represent all farms that were positive with FQ^R^ *Campylobacter*. All *C. coli* isolates harbored a single Thr-86-Ile mutation in GyrA without any other nucleotide changes in this region (Table 4). Among the 27 FQ^R^ *C. jejuni* isolates sequenced in this study, 10 isolates had the Thr-86-Ile point mutation only, 8 isolates carried the Thr-86-Ile mutation plus the Arg-285-Lys mutation or the Asn-203-Ser change, and 7 isolates carried theAsn-203-Ser and Arg-285-Lys mutations, one of which had an additional Ser-22-Gly change. Interestingly, 2 FQ^R^ *C. jejuni* isolates had no point mutations in QRDR. (Table 4, Table S1). Ser-22-Gly and Arg-285-Lys substitution have not been previously reported to be associated with FQ resistance in *Campylobacter*. However, Arg-285-Lys mutation was also found in ciprofloxacin susceptible isolates, indicating this point mutation alone would not confer FQ resistance.

**Table 4:** Point mutations observed in the QRDR of GyrA in FQ^R^ *C. jejuni* (n = 27) and *C. coli* (n = 27) isolates.

Species	Mutation(s)	Ciprofloxacin MIC (μg/ml)	No. of isolates
*C. jejuni*	Thr-86-Ile	8–32	10
	Thr-86-Ile Arg-285-Lys	4–64	5
	Asn-203-Ser Arg-285-Lys	8–64	6
	Thr-86-Ile Asn-203-Ser	16–32	3
	Ser-22-Gly Asn-203-Ser Arg-285-Lys	16	1
	NF	4–16	2
*C. coli*	Thr-86-Ile	8–16	27

NF: no point mutation found in gyrA.

Additionally, we analyzed the resistance determinant for tetracycline resistance using a *tet*(O)-specific PCR. Among the 20 tetracycline-resistant *Campylobacter* isolates examined in this study, all were positive with *tet*(O), indicating it is responsible for the resistance phenotype.

## Discussion

Results from this study revealed high prevalence of FQ^R^ *Campylobacter* in feedlot cattle in the U.S. The resistance rate in *C. coli* is especially high, reaching to 77%. Such a high-level prevalence of FQ resistance in ruminants was not reported in earlier studies conducted in the U.S.^19–21^, although a recent study conducted in 2008^25^ found that 27.3% *C. jejuni* and 49.2% *C. coli* from different types of cattle productions (including both feedlot cattle and adult cows) were resistant to ciprofloxacin. Our findings in this study showed an even higher frequency of resistance to ciprofloxacin (35.4% in *C. jejuni* and 77.3% in *C. coli*) and nalidixic acid (34.3% in *C. jejuni* and 82.6% in *C. coli*). All together, these observations clearly indicate a rising trend of FQ^R^ *Campylobacter* in ruminants in the U.S. The reason that FQ resistance was much more prevalent in *C. coli* than in *C. jejuni* is unknown, but it has been known that *C. coli* is more likely to acquire antibiotic resistance than *C. jejuni* ^23, 27–30^.

*Campylobacter* is highly mutable, and multiple independent studies including our work have demonstrated the rapid emergence of FQ^R^ mutants in animals originally infected with FQ^S^ *C. jejuni* and then treated with an FQ antimicrobial^2, 31–34^. FQ^R^ mutants spontaneously occur in *Campylobacter* populations and use of FQ antimicrobials selects and enriches these mutants. In *Campylobacter*, FQ-resistance is mainly mediated by point mutations in the QRDR of DNA gyrase (GyrA) in conjunction with the function of the multidrug efflux pump CmeABC^2, 26, 35, 36^. The most frequent mutation observed in FQ^R^ *Campylobacter* isolates is Thr-86-Ile, followed by Asp-90-Asn, Thr-86-Lys, Thr-86-Ala, Thr-86-Val, Asp-90-Tyr and Ala-70-Thr^2, 37, 38^. The Thr-86-Ile mutation confers a high level of FQ resistance (ciprofloxacin MIC ≥ 16 µl/ml) in *Campylobacter*, while other mutations are associated with a low to medium level of resistance (MIC = 1–8 µg/ml)^2, 39, 40^. Double mutations including Thr-86-Ile/Pro-104-Ser and Thr-86-Ile/Asp-90-Asn have also been linked to FQ resistance in *Campylobacter* ^39^.

Consistent with the previous findings discussed above, we found in this study that the FQ^R^ *C. coli* and *C. jejuni* isolates. However, we also identified three additional amino acid substitution in the *C. jejuni* isolates. One is Asn-203-Ser, which is known to confer FQ resistance along with Thr-86-Ile mutation^27, 41^. Another one is Arg-285-Lys, which alone may not confer FQ resistance because it was identified in susceptible strains. Six FQ^R^ contained both Asn-203-Ser and Arg-285-Lys, but no Thr-86-Ile (Table 4; Table S1). Whether the two mutations alone were responsible for the FQ resistance phenotype is unknown and needs further investigation. The third mutation is Ser-22-Gly, which has not been associated with FQ resistance in *Campylobacter*. Interestingly, two FQ^R^ *C. jejuni* isolates from the same farm did not show any mutations in QRDR of *gyrA* (Table 4), and what is responsible for their resistance to FQ is unknown and can’t be explained by mutations in *gyrA*. These findings indicate that the *gyrA* mutations in FQ^R^ *C. jejuni* isolates are more diverse than in FQ^R^ *C. coli* isolates.

FQ antibiotics are frequently used in veterinary medicine for the treatment and control of infectious diseases of pets and food-producing animals^42^. For example, enrofloxacin and danofloxacin are approved as an injectable solution (various dosage regimens) for use in the treatment and control of respiratory disease in cattle associated with *Mannheimia haemolytica*, *Pasteurella multocida*, *Histophilus somni* and *Mycoplasma bovis* in the United States and many other countries^42^. The initial approval of enrofloxacin by FDA was in 1998 and it was only for the treatment of bovine respiratory disease (BRD) in beef cattle. Subsequent approvals extended their use for BRD treatment in dairy replacement heifers of less than 20 months of age in 2008, and their metaphylactic use for control of BRD in beef and non-lactating dairy cattle at high risks of developing BRD in 2012. About 43% of the feedlots included in the Feedlot 2011 NAHMS study reported therapeutic use of FQs in approximately 42% of cattle with respiratory disease^43^. In the United States, the use of these drugs in cattle production is permitted only under a prescription from a veterinarian and their extralabel use in food producing animals is strictly prohibited. In this study, we showed a substantial increase in the prevalence of FQ resistance in *Campylobacter* isolates from cattle in the U.S., which coincides with the expanded use of FQ antibiotics in cattle production. However, it is still unknown if the on-farm use directly influences the development and dissemination of FQ-resistant *Campylobacter* and if the treatment regimen can be managed to reduce the development and prevalence of FQ-resistance.

Except for FQs and tetracycline, the *Campylobacter* isolates examined in this study are generally susceptible to other tested antimicrobials (Table 2). For example, the resistance to macrolide (erythromycin) was barely detected in both *C. coli* and *C. jejuni*. Tetracycline has been used for animal production for many years, and we found that tetracycline resistance is high in both *C. jejuni* (88.1%) and *C. coli* (74.8%), which is even higher than previously reported^19, 20, 44^. The predominant tetracycline resistance determinant in *Campylobacter* is *tet* (*O*), although a recent study reported that *tet* (*A*) also conferred resistant to tetracycline in *Campylobacter* ^45^. In this study, all examined tetracycline-resistant isolates harbored the *tet*(*O*) gene, consistent with previously reported findings.

PFGE and MLST are two commonly used genotyping methods for differentiation of *Campylobacter* isolates^46–48^. In this study, PFGE typing of 26 FQ^R^ *C. coli* and 24 FQ^R^ *C. jejuni* revealed that *C. coli* is more clonal than *C. jejuni* (Fig. 1), despite the fact that the *C. coli* isolates were from 15 feedlots in 5 different states. The majority of the PFGE-typed *C. coli* isolates were grouped into three clusters (I, II and IV) of high genetic similarity, which was confirmed by MLST to be a single ST (ST-1068), suggesting dissemination of a single clone on different cattle farms. This ST was observed in 83% (52/63) of the *C. coli* isolates of cattle origin in another report^49^, further indicating that it is highly prevalent in cattle. The presence of *C. coli* with identical genotype on multiple geographically distant farms implies the dissemination of a single strain from farm to farm, which could be a potential factor driving the increase in FQ^R^ prevalence in *Campylobacter*. Similar findings were reported in a previous study, in which multiple antibiotic resistant *C. coli* collected from different cattle farms and at different times had an indistinguishable PFGE pattern, in contrast to the genetic diverse of *C. jejuni* isolates^50^. The exact vehicles or mechanisms promoting clonal dissemination of *C. coli* in different cattle farms are unknown and need to be further investigated. For the C. *jejuni* isolates examined in this study, they are genetically diverse and it is unlikely that colonal expansion is involved in their dissemination.

In summary, we observed high prevalence of FQ-resistance in both *C. jejuni* and *C. coli* isolates derived from cattle in the U.S. The reason for this rising trend in FQ resistance is uncertain, but it is likely due to the hyper-mutable nature of *Campylobacter* and the selection from use of FQ antimicrobials in the control of respiratory diseases in cattle production. Additionally, clonal expansion, as reported in other studies^51–53^, may have also contributed to the increasing prevalence of FQ^R^ *Campylobacter*. Development of FQ resistance is known to affect the fitness of *Campylobacter* in chickens, resulting in persistence of FQ^R^ *Campylobacter* even in the absence of antibiotic selection pressure^54^.

Considering this possibility and the fact that FQs are currently used for cattle production, it is possible that the prevalence of FQ^R^ *Campylobacter* will continue to rise. Given that ruminant *Campylobacter* is a significant source of foodborne campylobacteriosis in humans, heightened efforts are needed to control the development and dissemination of FQ^R^ *Campylobacter* in cattle production.

## Methods and Materials

### Sample collection and bacterial isolation

A total of 3,184 cattle fecal samples were collected from 35 different feedlot cattle herds located in Iowa (n = 8), Texas (n = 8), Colorado (n = 8), Missouri (n = 3) and Kansas (n = 8) on two different occasions during December 2012 to March 2013. Collection of cattle fecal samples followed methods that have been described previously^55^. Cattle fecal samples were collected from the floor of animal pens and only freshly voided fecal pats were sampled. In other words, the sample was collected from a fecal pat only after a cow was observed defecating. This procedure allowed us to standardize environmental exposure time among fecal samples and estimate herd prevalence of *Campylobacter* without confining animals for collection of rectal samples. Freshly voided fecal pats were scraped with a sterile cotton tipped swab and the swab was immediately placed in 10 ml glass tubes containing *Campylobacter* Thioglycollate Broth (CAMPY-THIO). Vials were labeled and then immediately placed in an electric cooler set to 4 °C. All cattle fecal samples were shipped priority overnight to the testing laboratory. All samples were shipped, in insulated boxes packed with Ice-Brix® (Polar Tech Industries, Genoa, IL 60135). Only samples received by the laboratories within 24 hours of the date of collection were screened for *Campylobacter*. From the Campy-Thio containing the fecal samples, 1 ml was added into tubes containing 9 ml of *Campylobacter* enrichment broth (Mueller–Hinton [MH] medium supplemented with selective growth supplements [SR084E and SR117E; Oxoid]), and incubated at 42 °C for 48 h under microaerobic conditions (5% O_2_, 10% CO_2_, and 85% N_2_). From the enrichment culture, an inoculum of 100 µl was streaked onto MH agar containing the same selective supplements, which were further incubated for 48 h under the same conditions. A single *Campylobacter*-like colony from each sample was subcultured onto a MH agar plate and the pure cultures were stored in glycerol stocks at −80 °C until further use.

### *Campylobacter* identification

PCR was used to detect and differentiate *C. jejuni* and *C. coli*. Two sets of previously published PCR primers were used^56, 57^. The first primer pair (CCCJ-F: 5′-AAT CTA ATG GCT TAA CCA TTA-3′; CCCJ-R: 5′-GTA ACT AGT TTA GTA TTC CGG-3′), targeting 16S rRNA, was designed to co-identify *C. jejuni* and *C. coli* ^56^. The second primer pair (mapA-F: 5′-GAG TGC TTG TGC AAC TAA AC-3′; mapA-R: 5′-ATA GCA TCT TGA GTT GCT CC-3′) was specific for *C. jejuni* only^57^. The primers were synthesized at the DNA facility in Iowa State University using the MerMade-192 synthesizer. PCR reactions were performed as described previously^56, 57^.

### Antimicrobial susceptibility testing

In total, 320 *C. jejuni* (5 from each feedlot) and 115 *C. coli* (3 from each feedlot) isolates were randomly chosen and included in the susceptibility testing. The minimum inhibitory concentrations (MICs) of nine antibiotics were determined using a standard broth microdilution method as recommended by Clinical and Laboratory Standards Institute (CLSI) and National Antimicrobial Resistance Monitoring System for Enteric Bacteria (NARMS)^58–60^. Commercially available Sensititre *Campylobacter* plates (Trek Diagnostic Systems, Cleveland, Ohio) were used for antimicrobial susceptibility testing. The plates were read after incubated in a microaerobic environment for 24 h at 42 °C. The lowest antimicrobial concentration at which no bacterial growth developed was used as MIC value for each isolate. The antimicrobial resistance breakpoints were chosen according to the interpretive standards established by the CLSI for bacteria isolated from animals^58–60^. *C. jejuni* ATCC 33560 and *C. coli* ATCC 33559 were used as quality control strains for *C. jejuni* and *C. coli*, respectively.

### PFGE and MLST typing of *Campylobacter* isolates

In total, 26 FQ^R^ *C. jejuni* and 24 FQ^R^ *C. coli* isolates were randomly chosen from different feedlots in different states. In this study, 15 feedlots were positive with FQ^R^ *C. coli*, and 17 feedlots were positive with FQ^R^ *C. jejuni*. We selected 1–2 isolates from each positive feedlot to represent all positive farms in all five states. PFGE analysis of the macrorestriction fragment patterns of genomic DNA using KpnI enzyme was performed on these isolates following the CDC’s standardized PulseNet protocol for *Campylobacter* with minor modifications^47^. Briefly, fresh cultures of *Campylobacter* were embedded in 1% Seakem Gold agarose (Fisher Scientific, Fair Lawn, NJ) and lysed with proteinase K for 1 h at 55 °C in a water bath shaker. The gel plugs were digested with KpnI for 4 h at 37 °C. Digested plugs were embedded into 1% agarose and separated by electrophoresis in 0.5 × TBE buffer (Promega) at 14 °C for 18 h using a Chef Mapper electrophoresis system (Bio-Rad, Hercules, CA). Gel was stained with ethidium bromide for 30 min and then photographed by UV transillumination (Alpha Innotech, Santa, Clara, CA). The PFGE patterns were analyzed by the GelCompare II v.6.5 software program (Applied Maths, Kortrijik, Belgium) using Dice similarity coefficient and unweighted-pair group method with arithmetic averages (UPGMA) with 0.5% optimization and 1.5% position tolerance. Lambda DNA ladder (Bio-Rad) was used as the molecular size marker.

To further confirm the genotype of those isolates, MLST, originally developed by Dingle *et al*.^46^, was carried out on FQ^R^ *C. coli* and *C. jejuni* isolates representative of different PFGE types. Seven housekeeping genes were amplified and sequenced using the primer sets recommended at the *Campylobacter* MLST website (http://pubmlst.org/campylobacter/), developed by Keith Jolley and Man-Suen Chan at the University of Oxford^61^. All PCR products were purified using the QIAquick® PCR purification kit (QIAGEN, Hilden, Germany) and then sequenced at the DNA Core Facility of Iowa State University using an Applied Biosystems 3730xl DNA Analyzer. Allelic numbers were assigned to the isolates by performing BLAST searches for the assembled sequences using the single-locus query function, whereas sequence types were assigned using the allelic profile query function in the MLST database. Sequences that were identical to existing alleles in the MLST database were assigned the corresponding allele numbers. Novel sequences were assigned new allele numbers and sequence types (STs) within the MLST database.

### Antibiotic resistance mechanism determination

A total of 27 FQ^R^ *C. coli* and 27 FQ^R^ *C. jejuni* isolates were selected for determination of the point mutations in *gyrA* (Table S1). To amplify the QRDR region of *gyrA* by PCR, primers GyrAF1 (5′-CAACTGGTTCTAGCCTTTTG-3′) and GyrAR1 (5′-AATTTCACTCATAGCCTCACG-3′) were used for *C. jejuni*, while GyrAF2 (5′-TTATTTAGATTATTCTATGAGCGT-3′) and GyrAR2 (5′-CTTGAGTTCGATTACAACAC-3′) were used for *C. coli*. All PCR products were purified using the QIAquick® PCR purification kit (QIAGEN, Hilden, Germany) and then sequenced at the DNA Core Facility of Iowa State University using an Applied Biosystems 3730xl DNA Analyzer.

The presence of the *tet*(*O*) gene (the predominant determinant of tetracycline resistance in *Campylobacter*), was determined by PCR. For this purpose, primers *tet*(*O*)-F (5′-GGCGTTTTGTTTATGTGCG-3′) and *tet*(*O*)-R (5′-ATGGACAACCCGACAGAAGC-3′) were used to amplify a 559-bp region of the *tet*(*O*) gene as described elsewhere^47^.

### Statistical analysis

To compare the prevalence of antimicrobial resistance between *C. jejuni* and *C. coli*, the statistical analyses were performed with GLIMMIX procedure in SAS 9.4 version (SAS Institute Inc., Cary, NC, USA) for binary distribution (yes/no response variable) with logit link function. Both states and farms were considered as random effects, the *Campylobacter* species (*C. jejuni* and *C. coli*) was the fixed effect, and three different models were fitted separately with three kinds of antibiotics (ciprofloxacin, nalidixic acid and tetracycline) as response variables. The significance level used here is 0.05.

## Electronic supplementary material

Supplementary information
