# Supplementary material for: Rising fluoroquinolone resistance in Campylobacter isolated from feedlot cattle in the United States
Source: Sci Rep. 2017 Mar 29;7:494. doi: 10.1038/s41598-017-00584-z (PMC5428712; doi:10.1038/s41598-017-00584-z)
Supplement: Supplementary file 1 — Supplementary information [file 41598_2017_584_MOESM1_ESM.pdf]

Rising fluoroquinolone resistance in *Campylobacter* isolated from feedlot cattle in the United States.

Yizhi Tang[1], Orhan Sahin[1,2]*, Nada Pavlovic[1], Jeff LeJeune[3], James Carlson[4], Zuowei Wu[1], Lei Dai[1] and Qijing Zhang[1]*

[1]Department of Veterinary Microbiology and Preventive Medicine, and [2]Department of Veterinary Diagnostic and Production Animal Medicine, Ames, IA, USA; [3]Food Animal Health Research Program, Ohio State University, Wooster, OH; [4]National Wildlife Research Center, USDA APHIS, Fort Collins, CO.

*Send correspondence to osahin@iastate.edu, or zhang123@iastate.edu

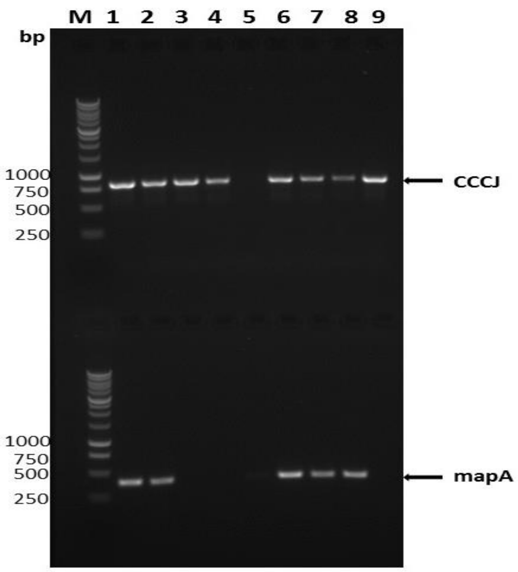

Figure. S1. Representative gel electrophoresis images for the identification of *C. jejuni* and *C. coli* using PCR. The upper panel shows the PCR product amplified by the CCCJ primer pair which identifies both *C. jejuni* and *C. coli*, while the lower panel shows the PCR product amplified by the *mapA* primer pair, which is specific for *C. jejuni*. M: maker, 1-9: different *Campylobacter* isolates including 5 *C. jejuni* isolates (lane 1, 2, 6-8), 3 *C. coli* isolates (lane 3, 4 and 9) and one untypeable isolate (lane 5).

Table.S1. The sources of FQ^R *C. jejuni* (n = 27) and *C. coli* (n = 27) isolates tested for *gyrA* mutations

Species	Isolate ID	State	Mutation(s)	Ciprofloxacin MIC (μg/ml)
C. jejuni	TX-1-FC-81	Texas	Thr-86-Ile Arg-285-Lys	4
	TX-2-FC-64	Texas	Asn-203-Ser Arg-285-Lys	16
	TX-4-FC-40	Texas	Thr-86-Ile	8
	TX-5-FC-27	Texas	Thr-86-Ile Arg-285-Lys	64
	TX-7-FC-83	Texas	Thr-86-Ile	8
	TX-8-FC-22	Texas	Thr-86-Ile Arg-285-Lys	16
	KS-1-FC-53	Kansas	Thr-86-Ile	32
	KS-3-FC-33	Kansas	Thr-86-Ile Asn-203-Ser	32
	KS-4-FC-80	Kansas	Thr-86-Ile	8
	KS-5-FC-30	Kansas	Thr-86-Ile	16
	KS-8-FC-6	Kansas	Thr-86-Ile Asn-203-Ser	16
	CO-8-FC-1	Colorado	Thr-86-Ile	8
	CO-1-FC-22	Colorado	Ser-22-Gly Asn-203-Ser Arg-285-Lys	16
	CO-2-FC-1	Colorado	Asn-203-Ser Arg-285-Lys	64
	CO-3-FC-92	Colorado	Asn-203-Ser Arg-285-Lys	8
	CO-4-FC-1	Colorado	Thr-86-Ile	16
	CO-5-FC-26	Colorado	Thr-86-Ile	16
	IA-3-FC-18	Iowa	Not detected	16
	IA-6-FC-93	Iowa	Not detected	4
	IA-5-FC-42	Iowa	Thr-86-Ile Asn-203-Ser	16
	IA-4-FC-75	Iowa	Asn-203-Ser Arg-285-Lys	16
	IA-4-FC-1	Iowa	Asn-203-Ser Arg-285-Lys	16
	IA-8-FC-99	Iowa	Asn-203-Ser Arg-285-Lys	8
	MO-1-FC-27	Missouri	Thr-86-Ile	16
	MO-2-FC-1	Missouri	Thr-86-Ile Arg-285-Lys	8
	MO-2-FC-9	Missouri	Thr-86-Ile Arg-285-Lys	16
	MO-3-FC-95	Missouri	Thr-86-Ile	8

C. coli	KS-2-FC-11	Kansas	Thr-86-Ile	16
	KS-4-FC-86	Kansas	Thr-86-Ile	16
	KS-5-FC-66	Kansas	Thr-86-Ile	8
	KS-6-FC-13	Kansas	Thr-86-Ile	8
	KS-8-FC-18	Kansas	Thr-86-Ile	8
	KS-1-FC-100	Kansas	Thr-86-Ile	16
	KS-2-FC-7	Kansas	Thr-86-Ile	16
	TX-7-FC-38	Texas	Thr-86-Ile	16
	TX-6-FC-7	Texas	Thr-86-Ile	16
	TX-8-FC-17	Texas	Thr-86-Ile	8
	TX-1-FC-85	Texas	Thr-86-Ile	8
	TX-5-FC-88	Texas	Thr-86-Ile	16
	CO-6-FC-3	Colorado	Thr-86-Ile	8
	CO-7-FC-50	Colorado	Thr-86-Ile	4
	CO-1-FC-54	Colorado	Thr-86-Ile	16
	CO-4-FC-51	Colorado	Thr-86-Ile	8
	CO-5-FC-44	Colorado	Thr-86-Ile	8
	CO-1-FC-88	Colorado	Thr-86-Ile	16
	MO-1-FC-43	Missouri	Thr-86-Ile	16
	MO-1-FC-26	Missouri	Thr-86-Ile	8
	MO-1-FC-58	Missouri	Thr-86-Ile	16
	MO-2-FC-2	Missouri	Thr-86-Ile	8
	IA-6-FC-48	Iowa	Thr-86-Ile	8
	IA-3-FC-17	Iowa	Thr-86-Ile	16
	IA-5-FC-41	Iowa	Thr-86-Ile	16
	IA-4-FC-3	Iowa	Thr-86-Ile	8
	IA-1-FC-89	Iowa	Thr-86-Ile	8
	IA-5-FC-50	Iowa	Thr-86-Ile	16
